# The Cost of Provisions in Institutions.—VI

**Published:** 1904-05-21

**Authors:** 


### HOSPITAL ADMINISTRATION.

### CONSTRUCTION AND ECONOMICS.

### THE COST OF PROVISIONS IN INSTITUTIONS.—VI.

By the Author of "Hospital Expenditure—The Commissariat."

(*Continued from page* 123.)

GUY'S HOSPITAL (Available Beds, 652).

The cost of provisions at Guy's Hospital for the last five years has been as follows:—

| Year | Daily average of occupied beds | Average cost of provisions per bed |
|---|---|---|
| | | £ |
| 1902 ... ... ... | 485 | 29¼ |
| 1901 ... ... ... | 470 | 28¼ |
| 1900 ... ... ... | 464 | 26¾ |
| 1899 ... ... ... | 458 | 25 |
| 1898 ... ... ... | 424 | 25½ |

The statement of expenditure on provisions furnished in the report of this institution is exceptionally interesting and valuable for purposes of comparison. It is divided into three sections. The first, which is given in detail, relates solely to the cost of the patients. Under the second heading is the amount paid out for the board of the resident medical staff. Lastly, the board of the nursing staff, female servants, and wardmaids is entered. We get the following figures:—

| Year | Number of occupied beds out of 625 available | Total cost of provisions | Cost of patients at £16 a year | Average cost of provisions, exclusive of patients | |
|---|---|---|---|---|---|
| | | | | (*a*) per occupied bed | (*b*) per occupied bed |
| 1902 ... | 485 | £ 14,251 | £ 7,815 | £ 13 | £ 9¾ |

The number of those boarded in the institution, other than patients, is 39 per cent. of the total inmates, as follows:—

| | Number | Average of available beds to each | Average of occupied beds to each |
|---|---|---|---|
| Medical officers ... | 17 | 38 | 28 |
| Nursing staff ... | 234 | 2·7 | 2 |
| Female servants ... | 26 | 25 | 19 |
| Wardmaids ... | 40 | 16 | 12 |
| Total ... ... | 317 | 2 | 1·5 |

The board of about thirty laundry-maids is included under the head of washing.

Taking the patients first, it will be interesting to see the exact cost of each item of their board as detailed in the accounts. The average cost is almost exactly £16 a year, 6s. 1¼d. a week, or 10½d. a day.

*Cost of Patients.*

| | Total cost | Cost per bed, 485 occupied |
|---|---|---|
| | £ | £ s. d. |
| Meat ... ... ... ... | 2,247 | 4 12 0 |
| Fish, poultry, etc. ... ... | 994 | 2 0 0 |
| Butter, cheese, etc. ... ... | 467 | 0 19 0 |
| Eggs ... ... ... ... | 418 | 0 17 0 |
| Milk ... ... ... ... | 1,532 | 3 3 0 |
| Bread, flour, etc. ... ... | 601 | 1 4 0 |
| Grocery ... ... ... | 1,304 | 2 13 0 |
| Vegetables ... ... ... | 227 | 0 9 0 |
| Malt liquors ... ... ... | 20 | 0 0 11 |
| Ice and mineral waters ... | 296 | 0 12 0 |
| Wine and spirits ... ... | 395 | 0 16 0 |

These figures include also the cost of Bright's Ward, where paying patients are received, and the board is of a somewhat more liberal description. As, however, there were only 411 paying patients in 1902, out of a total of 8,091 in-patients, the distinction, although considerable in individual comparison, is not important in examining the averages in detail. The cost of boarding the 411 paying patients amounted to £873, or an average of £2 2s. a head, whereas the cost of boarding the ordinary patient works out at about 18s. a head, including, in both cases, the whole time of residence in the hospital. Deducting the cost of provisions for Bright's Ward and the average number of beds occupied in it from the daily average of occupied beds, it will be found that the average cost of provisions per bed is lowered to £15 a year, 5s. 9¼d. a week, or 9¾d. a day.

The average weight of meat consumed by the patients is about 3½ lb. a week, or ½ lb. a day, uncooked, as received from the butcher.

On a given day 330 quarts of milk were consumed by the patients. Taking the whole year, it would appear that a daily average of about 460 quarts for the patients, or nearly a quart a day each.

The average number of patients on milk diet is 80. Full diet consists of bread 12 oz., butter 1 oz., milk 1 pint, potatoes 8 oz., cooked meat 6 oz., in addition to half a pint of mutton broth, or half a pound of rice pudding, tea and sugar. Milk diet consists of 3 pints per diem. A variation of milk diet known as "farinaceous" carries 2 pints of milk, 12 oz. of bread, 1 oz. butter. There are the usual special diets, including a regular diabetic diet.

The cost of the medical officers for board is £75 a year each. This amount is paid out for their board, and includes, it may be remarked, the entire expense not only of the uncooked food, but of wages, service, rent, fuel, lighting, etc. The difference in cost of providing board in the institution and of paying for it to be provided elsewhere is not always appreciated. If the accommodation, service, etc., are added to the actual cost of the food, it will often be found to amount to as much as 5s. per week per head, only the items, being otherwise distributed in the accounts, do not in this case make themselves felt in the cost of provisions.

The nursing staff, female servants, and wardmaids make up a total of 300, boarded at a total cost of £5,162, or an average of £17 4s. a head.

There is an additional sum of £412 which represents the cost of about 24 nurses in training contributed by the nursing institution. In analysing the cost of each inmate under different headings, we have been compelled to include this amount, and have estimated the number of persons boarded under this account at 324 (see table opposite).

It will be noticed that the average cost of the household for bread is unusually low—17s. as compared with 24s. for the patients. The explanation of this apparent anomaly lies in the fact that there is a private bakery in the institution, where not only all the ordinary breadstuffs are made, but also all the diabetic preparations—usually a very costly item not included under bread and flour. Hence a great economy is effected in the bread bill, and a still greater one in the diabetic preparations.

The mineral waters are made in the hospital, but n[illegible] the ice.

| | Cost of provisions | Average per head |
|---|---|---|
| | £ | £ s. |
| Meat ... ... ... ... | 1,542 | 4 15 |
| Fish, poultry, etc. ... ... | 293 | 0 18 |
| Butter, cheese, etc. ... ... | 1,381 | 4 5 |
| Eggs ... ... ... ... | 130 | 0 8 |
| Milk ... ... ... ... | 531 | 1 12 |
| Bread, etc. ... ... ... | 283 | 0 17 |
| Grocery ... ... ... ... | 793 | 2 7 |
| Vegetables ... ... ... | 461 | 1 8 |
| Malt liquors ... ... ... | 77 | 0 4 |

